# Bilateral cleft lip repair with simultaneous premaxillary setback and primary limited rhinoplasty

**DOI:** 10.1186/s40902-018-0182-0

**Published:** 2018-12-20

**Authors:** Young-Wook Park, Chan-Woo Kim

**Affiliations:** Department of Oral and Maxillofacial Surgery, College of Dentistry, Gangneung-Wonju National University, 7 Jukheon-Gil, Gangneung, Gangwondo 25457 Korea

**Keywords:** Bilateral cleft lip and palate, Vomerine ostectomy, Premaxillary setback, Primary rhinoplasty

## Abstract

**Background:**

Functional closure of the orbicularis oris muscle and esthetic reconstruction of nasolabial components are impossible in patients with severely deformed premaxilla. Here, we review a surgical strategy for patients with unremedied premaxilla retrospectively.

**Results:**

Vomerine ostectomy and premaxillary setback with nasolabial repair were performed in 12 patients with bilateral cleft lip and palate. The mean age of patients was 21.7 months. The extent of ostectomy varied between 3 and 11 mm. There were no serious complications from defective perfusion to the premaxilla or the philtral flap. The follow-up period ranged from 2 to 25 months. Proper positioning of the premaxilla and satisfactory nasolabial esthetics were achieved in all patients.

**Conclusions:**

We performed nasolabial repair after premaxillary setback without jeopardizing the premaxillary segment or the philtral flap. Our surgical strategy could be recommended in poor socio-economic circumstances due to the cost effectiveness of limiting the number of surgeries.

## Background

Protruding or rotating premaxilla is a common feature in patients with bilateral cleft lip and palate (BCLP). It is due to the unrestrained growth at anterior nasal septal and vomero-premaxillary suture (VPS), without lateral continuities [1]. In these circumstances, functional closure of the orbicularis oris muscle and esthetic reconstruction of the nasolabial components are impossible during primary cheiloplasty.

To align the premaxillary segment with lateral alveolar segments, several dentofacial orthopedic devices were used [2–4]. In some cases, however, there were no responses from the orthopedic forces, especially in patients with age greater than 10 months [5]. Furthermore, lots of patients are precluded from preoperative dentofacial orthopedics due to poor socio-economic environments. In such cases, vomerine ostectomy and premaxillary setback may be useful for surgical repair of BCLP.

In this study, we present our surgical concept and modality in patients with severely deformed premaxillae of BCLP. In our practice, we also perform primary rhinoplasty although it might compromise prolabial perfusion. We determined whether the combined premaxillary setback and nasolabial repair causes deflective circulation to the premaxillary segment or the prolabial flap.

## Methods

Institutional review board of Gangneung-Wonju National University Dental Hospital approved this retrospective study (IRB No. 2016-015) for patients with BCLP. Inclusion criteria were as follows: nonsyndromic bilateral cleft lip and palate, the patients who had not underwent previous dentofacial orthopedic treatments, and the distance between the central and the lateral alveolar segments was over 5 mm on either side. Surgeries were performed by one surgeon between 2015 and 2017. Data were obtained from medical records and perioperative photographs. The surgical techniques were as follows. Under general anesthesia, primary cheiloplasty was performed concomitantly with premaxillary setback and primary rhinoplasty. Through a midline incision on the inferior border of the vomer, minimal amount of the bone was removed using a bone rongeur, anterior to the VPS, when the premaxilla was predominantly rotating. After bending the premaxilla, the wound was approximated without interosseous fixation. However, when the premaxilla was predominantly protruding, the required bone was removed posterior to the VPS. After premaxillary setback, it was stabilized via suture material or titanium plate and screws. After premaxillary repositioning, we established orbicularis muscular continuity and philtral flap preparation, which was narrow enough to meet the modern surgical concept of bilateral cleft lip repair [6]. Finally, the flattened nasal cartilages were exposed via nostril-rim or reverse U-shaped incision. For all patients, lower lateral cartilages of the nose were approximated using two or three transfixation sutures with 5–0 PDS.

## Results

Twelve patients with BCLP, who showed severely deformed premaxilla, were included in this study. Eight patients were diagnosed as complete BCLP and four patients were diagnosed as unsymmetrical BCLP. The ages of patients at the time of premaxillary setback ranged from 5 months to 8 years and 1 month. All patients had not undergone any type of dentofacial orthopedic treatment, except labial taping.

The extent of vomer ostectomy varied ranging from 3 to 11 mm. For five patients with predominently rotating premaxilla, the wedge-shaped bone was removed anterior to the VPS. For seven patients with predominently protruding premaxilla, the required amount of the bone was removed in a rectangular shape. And the premaxillary segment was bodily repositioned. For two patients aged of 5 and 8 years, osteofixation was performed using a titanium plate.

Postoperatively, all patients were recovered uneventfully, and had no serious complications from defective perfusion (Table 1). However, one patient (number 7) showed partial dehiscence on the tip of the philtral flap, healed by scar tissue. The follow-up period ranged from 2 to 25 months. During the follow-up periods, all patients showed no signs of anterior cross-bite. Proper positioning of the premaxilla and satisfactory nasolabial esthetics were achieved in all patients.

**Table 1:** Clinical information of patients

Number	Age (Months)	Sex	Premaxillary status	Location of ostectomy in relation to VPS	Method of fixation	Incision for rhinoplasty	Follow-up period (months)
1	10	F	Predominantly rotating	Anterior	None	Nostril rim	25
2	11	F	Predominantly protruding	Posterior	Suture material	Inverted-U	12
3	14	F	Predominantly protruding	Posterior	Suture material	Inverted-U	12
4	12	M	Predominantly protruding	Posterior	Suture material	Inverted-U	15
5	97	M	Predominantly protruding	Posterior	Titanium plate	Nostril rim	25
6	8	M	Predominantly protruding	Posterior	Suture material	Inverted-U	12
7	8	M	Predominantly rotating	Anterior	None	Nostril rim	12
8	14	M	Predominantly rotating	Anterior	None	Via philtral flap	24
9	5	F	Predominantly rotating	Anterior	None	Nostril rim	2
10	8	M	Predominantly rotating	Anterior	None	Nostril rim	12
11	14	F	Predominantly protruding	Posterior	Suture material	Nostril rim	12
12	62	F	Predominantly protruding	Posterior	Titanium plate	Nostril rim	2

*VPS*= vomero-premaxillary suture

### Case 1: patient 4

**Fig. 1:**
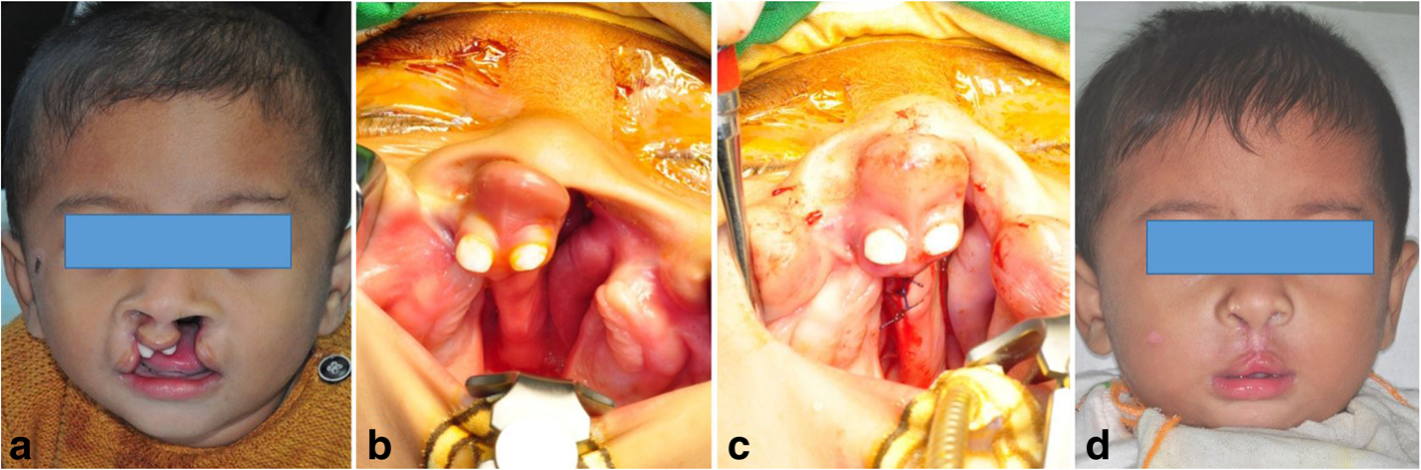
Case 1. **a**, **b** A 12-month-old male with unsymmetrical bilateral cleft lip and palate showed a predominently protruding premaxilla. **c** Repositioned premaxilla by vomerine ostectomy, posterior to the VPS. **d** Postoperative frontal photogram after concomitant premaxillary setback and nasolabial repair

A 12-month-old male patient with a predominently protruding premaxilla due to unsymmetrical BCLP was referred. To align the premaxilla, minimal amount (3 mm) of the bone was removed from the vomer, posterior to the VPS. The septo-premaxillary ligament anterior to the VPS was preserved. The repositioned premaxilla was stabilized by interosseous suturing with 2–0 polyglactin 910 (Vicryl®, Ethicon Inc., USA). After orbicularis muscular repair, limited rhinoplasty was performed via reverse U-shaped incision. Postoperatively, his columella was elongated, and nasolabial structures were normalized (Fig. 1).

### Case 2: patient 1

**Fig. 2:**
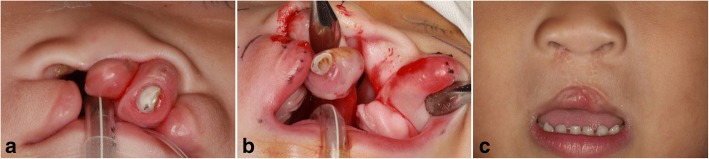
Case 2. **a** A 10-month-old female with bilateral cleft lip and palate showed a predominently rotating premaxilla. **b** Repositioned premaxilla by vomerine ostectomy, anterior to the VPS. **c** Frontal photogram 2 years after concomitant premaxillary repositioning and nasolabial repair

A 10-month-old female patient with a predominently rotating premaxilla due to a complete BCLP was referred. To align the premaxilla, minimal amount of the bone was removed from the vomer, anterior to the VPS. The premaxillary segment was indirectly stabilized with bilateral mucosal bridging over the alveolar gap. For this patient, the lower lateral cartilages of the nose were approximated via nostril rim incision. Two years later, her nasolabial structures were normalized and there were no signs of anterior crossbite (Fig. 2**.**)

### Case 3: patient 5

**Fig. 3:**
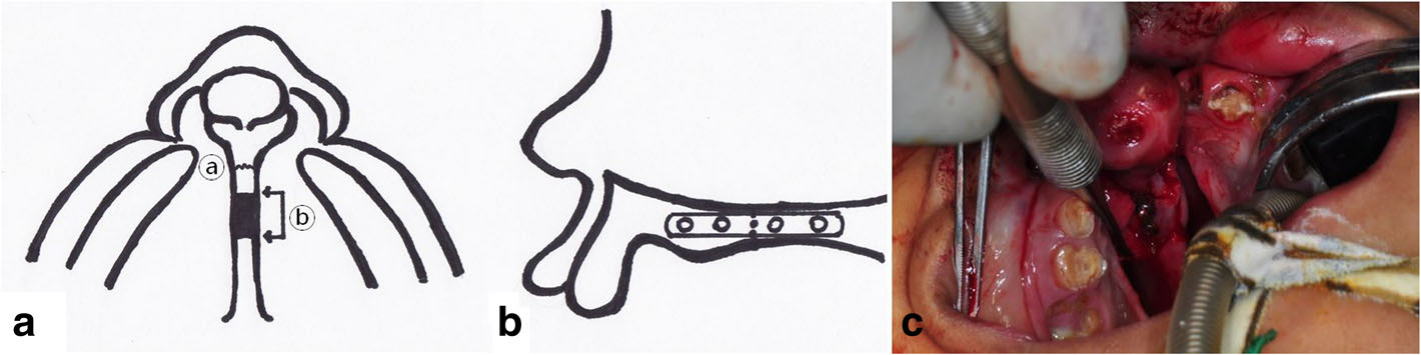
**a** Illustration of the osteotomy lines, ⓐ vemero-premaxillary suture, ⓑ osteotomy lines. **b** Illustration of osteofixation using a titanium plate after premaxillary setback. After two osteotomies posterior to VPS, we separated the lower edge of the septal cartilage from the vomer groove. **c** Intraoperative view of the titanium plate-osteofixation for the mobile premaxillary segment

**Fig. 4:**
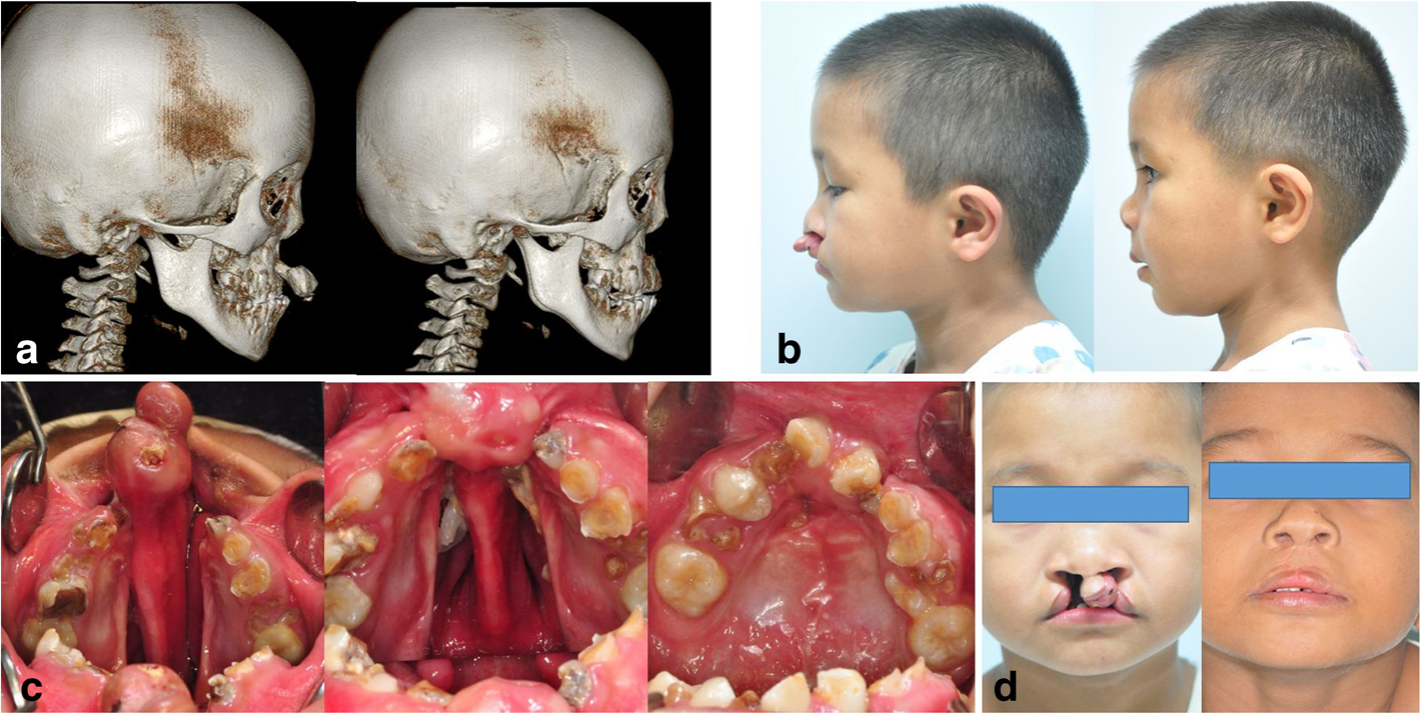
Case 3. **a** Preoperative and postoperative three-dimensional CT of an 8-year-old boy with a protruding premaxilla. **b** Preoperative and postoperative lateral photogram after concomitant premaxillary setback and nasolabial repair. **c** Mirror images of upper dental arch: preoperative, before palatoplasty, 2 years after the operation before alveolar bone grafting. **d** Preoperative and 2-year postoperative frontal photogram

An 8-year and 1-month-old male patient with protruding premaxilla due to BCLP was referred. To align the premaxilla, 11 mm of the bone was removed from the vomer, posterior to the VPS. The extent of ostectomy was determined by prediction tracing of the lateral cephalogram with consideration of postoperative ideal nasolabial angle. After separating the septal cartilage from the vomer groove, the premaxillay segment was bodily repositioned to its new position (back and up), where rigid fixation was performed using a 1.6-mm, 4-holed titanium plate and screws (M3®, Osteomed Co, U.S.A.) (Fig. 3). Labial repair and concomitant rhinoplasty were performed. Preoperative and postoperative three-dimensional CT images are also presented in Fig. 4. Two years later, his nasolabial structures were normalized, and his upper dental arch was well aligned without anterior crossbite (Fig. 4).

## Discussion

The repair of bilateral cleft lip nasal deformity remains to be a challenge for cleft surgeons especially when the premaxilla is severely deformed. There are so many patients in which dentofacial orthopedics are unsuccessful or unavailable. For these patients, surgical premaxillary setback might be considered for functional cheiloplasty. We have successfully performed nasolabial repair concomitantly with surgical setback of the premaxilla in selected patients with BCLP. Our surgical modality appears to reduce the social stigma of cleft face from the earliest time in the patient’s life.

Surgical premaxillary repositioning has been successfully combined with palatoplasty [7, 8] or alveolar bone grafting [9, 10]. However, the possible problem of this surgical maneuver is premaxillary perfusion. It is important to preserve blood supply from the soft tissue and periosteum of anterior premaxilla, as well as the nasal septum. According to the empirical principles of cleft surgery, philtral flap should not be elevated because vomerine ostectomy might abrogate the circulation from the mucoperiosteum of the nasal septum. But, after Cronin’s suggestion [11], modern precise surgery permitted synchronous philtral flap with vomerine ostectomy.

Primary one-stage cleft lip and nose repair in BCLP is a common tenet in modern cleft surgery [12, 13]. Fakih-Gomez reported four cases of vomerine ostectomy for premaxillary setback in bilateral cleft patients. However, they did not perform any primary nasal correction for fear of increased risk of impairment of the already compromised vascularity of the philtrum and premaxilla due to the vomerine ostectomy [14]. But, in our practice, primary rhinoplasty was safely combined with premaxillary setback and cheiloplasty. We tried to preserve the circulation from surrounding soft tissues using minimal incision for vomerine ostectomy and for primary rhinoplasty, followed by considerable surgical handling of labial tissue components. As a result, in our series of BCLP patients, there were no serious complications of premaxillary or prolabial ischemic necrosis. Only one patient, in which vomerine ostectomy, anterior to the VPS and nostril-rim incision for rhinoplasty had been performed, showed partial necrosis of the tip of the philtral flap that was healed by scar without significant deformity.

Vomero-premaxillary suture has been considered as a growth site for mid-facial development [15, 16]. Therefore, surgical trauma to VPS would be likely to cause impaired mid-facial growth in patients with BCLP. Vyas and Bittermann documented early occlusal signs of disturbed maxillary growth after primary premaxillary ostectomy and setback before the age of 2 years [5, 17]. On the contrary, Padwa concluded that premaxillary ostectomy in childhood does not further inhibit mid-facial growth [18]. Therefore, the role of vomerine ostectomy on mid-facial growth remains unclear.

In this study, the follow-up period was not enough to evaluate the mid-facial change after surgical premaxillary setback. In our patient group, 11 out of 12 were over the age of 7 months. Some surgeons recommended combined palatoplasty with premaxillary setback prior to cheiloplasty in case of late referral [5]. But, we performed combined nasolabial repair with premaxillary setback prior to palatoplasty for early improvement of childhood psychosocial and esthetic impact. During the follow-up period, we did not find any occlusal signs of mid-facial underdevelopment. We need to confirm the effect of primary premaxillary setback on mid-facial growth for a longer period. However, the well-known effect of palatoplasty on maxillary growth will be added. Practically, it is very difficult to evaluate the effect of primary premaxillary setback on mid-facial growth in patients with BCLP.

We tried to avoid direct surgical injury to the VPS by putting the osteotomy line anterior or posterior to VPS. Some surgeons performed vomerine ostectomy anterior to VPS [5, 14], while others recommended to put the osteotomy line posterior to VPS [8, 19]. In our practice, we put the osteotomy line anterior to VPS when a bodily movement of the premaxillary segment was not needed to avoid surgical maneuver of the septal cartilage. From the perspective of surgical trauma, the location of the osteotomy line to VPS does not seem to make much of a difference.

Repair of bilateral cleft lip concomitant with premaxillary setback and primary rhinoplasty can be considered to be a risky surgical option with respect to perfusion to the surgical site. However, we performed this procedure without jeopardizing the premaxillary segment or the philtral flap. As alluded to earlier, the authors think that the advantages of our surgical modality to be as follows: it permits functional closure of nasolabial muscles for cheilorhinoplasty, it permits following palatal and alveolar repair, and it permits early improvement of cleft appearance. For the possible disadvantage of mid-facial underdevelopment, it may be important to note that most patients with BCLP need mid-facial advancement after palatoplasty.

## Conclusions

Repair of bilateral cleft lip concomitant with premaxillary setback and primary rhinoplasty was successfully performed without jeopardizing the premaxillary segment or the philtral flap. Therefore, this can be a useful strategy for patients with BCLP, who cannot take advantage of social care, or failed to dentofacial orthopedic treatments. We need to wait to check whether this procedure inhibits the mid-facial growth.

## Abbreviation

No: Number

## References

[1] Latham RA (1973). Development and structure of the premaxillay deformity in bilateral cleft lip and palate. Br J Plast Surg.

[2] Meazzini M, Lematti L, Mazzoleni F, Rabbiosi D, Bozzetti A, Brusati R (2010). Vertical excess of the premaxilla in bilateral cleft lip and palate patients. J Craniofac Surg.

[3] Mahmood R, Flood T, Robinson S, Al-Gholmy M (2016). Early orthopedic retraction of the premaxilla in bilateral complete cleft lip and palate: an innovative approach to a difficult problem. Cleft Palate Craniofac J.

[4] Vura N, Gaddipati R, Palla Y, Kumar P (2018). An intraoral appliance to retract the protrusive premaxilla in bilateral cleft lip patients presenting late for primary lip repair. Cleft Palate Craniofac J.

[5] Vyas RM, Kim DC, Padwa BL, Mullikan JB (2016). Primary premaxillary setback and repair of bilateral complete cleft lip: indications, technique, and outcomes. Cleft Palate-Craniofac J.

[6] Mulliken JB (2000). Repair of bilateral complete cleft lip and nasal deformity—state of the art. Cleft Palate Craniofac J.

[7] Narayanan RK, Hussain SR, Murukesan S, Murthy J (2006). Synchronous palatal closure and premaxillary setback in older children with bilateral complete cleft of lip and palate. Plast Reconstr Surg.

[8] Murthy J (2009). Primary bilateral cleft lip repair with management of premaxilla without preoperative orthopedics. J Craniofac Surg.

[9] Burezq H, Bang RL, George A, Mukhtar A (2007). Unusual clinical presentation of a severely protruding premaxilla in patients with bilateral clefts. J Craniofac Surg.

[10] Rahpeyma A, Khajehahmadi S, Ghaseme A (2016). Premaxillary osteotomy fixation in bilateral cleft lip/palate: introducing a new technique. Asian J Surg.

[11] Cronin TD (1956). Management of bilateral cleft lip with protruding premaxilla. Am J Surg.

[12] Trott JA, Mohan N (1993). A preliminary report on one stage open tip rhinoplasty at the time of lip repair in bilateral cleft lip and palate: the Alor Setar experience. Br J Plast Surg.

[13] Xu H, Salyer KE, Genecov ER (2009). Primary bilateral one-stage cleft lip/nose repair: 40-year Dallas experience: part I. J Craniofac Surg.

[14] Fakih-Gomez N, Sanchez-Sanchez M, Iglesias-Martin F, Garcia-Perla-Garcia A, Belmonte-Caro R, Gonzalez-Perez LM (2015). Repair of complete bilateral cleft lip with severely protruding premaxilla performing a premaxillary setback and vemerian ostectomy in one stage surgery. Med Oral Patol Oral Cir Bucal.

[15] Friede H, Morgan P (1976). Growth of the vomero-premaxillary suture in children with bilateral cleft lip and palate. A histological and roentgencephalometric study. Scand J Plast Reconstr Surg.

[16] Friede H (1978). The vomero-premaxillary suture- a neglected growth site in mid-facial development of unilateral cleft lip and palate patients. Cleft Palate J.

[17] Bittermann GKP, de Ruiter AP, Janssen NG, Bittermann AJN, van der Molen AM, van Es RJJ, Rosenberg AJWP, Koole R (2016). Management of the premaxilla in the treatment of bilateral cleft of lip and palate: what can the literature tell us. Clin Oral Invest.

[18] Padwa BL, Sonis A, Bagheri S, Mulliken JB (1999). Children with repaired bilateral cleft lip/palate: effect of age at premaxillary osteotomy on facial growth. Plast Reconstr Surg.

[19] Kobayashi S, Hirakawa T, Fukawa T, Satake T, Maegawa J (2017) Synchronous premaxillay osteotomy with primary cheiloplasty for BCLP patients with protrusion of premaxillae. PRS Global Open. 10.1097/GOX.0000000000001402 10.1097/GOX.0000000000001402 PMC5732650 29263944

